# Asthma control factors in the Gulf Cooperation Council (GCC) countries and the effectiveness of ICS/LABA fixed dose combinations: a dual rapid literature review

**DOI:** 10.1186/s12889-020-09259-3

**Published:** 2020-08-08

**Authors:** Saeed Noibi, Ahmed Mohy, Raef Gouhar, Fadel Shaker, Tamara Lukic, Hamdan Al-Jahdali

**Affiliations:** 1 Medical Affairs Department, GSK Saudi Arabia, 22nd Floor Head Quarters Business Park, Jeddah, Kingdom of Saudi Arabia; 2 Medical Affairs Department, GSK Gulf Countries, Arenco Towers, Dubai Medial City, Dubai, United Arab Emirates; 3 King Saud bin Abdulaziz University for Health Sciences I KSAU-HS, College of Medicine, Riyadh, Kingdom of Saudi Arabia

**Keywords:** Asthma control factors, Effectiveness studies, ICS/LABA FDC, Gulf cooperation council (GCC) countries, Clinical practice, Rapid review, Evidence-informed policy-making

## Abstract

**Background:**

Asthma control is influenced by multiple factors. These factors must be considered when appraising asthma interventions and their effectiveness in the Gulf Cooperation Council (GCC) countries (Bahrain, Kuwait, Oman, Qatar, Saudi Arabia and United Arab Emirates [UAE]). Based on published studies, the most prevalent asthma treatment in these countries are fixed dose combinations (FDC) of inhaled corticosteroid and long-acting beta-agonist (ICS/LABA). This study is a rapid review of the literature on: (a) factors associated with asthma control in the GCC countries and (b) generalisability of ICS/LABA FDC effectiveness studies.

**Methods:**

To review local factors associated with asthma control and, generalisability of published ICS/LABA FDC studies, two rapid reviews were conducted. Review 1 targeted literature pertaining to asthma control factors in GCC countries. Eligible studies were appraised, and clustering methodology used to summarise factors. Review 2 assessed ICS/LABA FDC studies in conditions close to actual clinical practice (i.e. effectiveness studies). Eligibility was determined by reviewing study characteristics. Evaluation of studies focused on randomised controlled trials (RCTs). In both reviews, initial (January 2018) and updated (November 2019) searches were conducted in EMBASE and PubMed databases. Eligible studies were appraised using the *Critical Appraisal Skills Program (CASP) checklists*.

**Results:**

We identified 51 publications reporting factors associated with asthma control. These publications reported studies conducted in Saudi Arabia (35), Qatar (5), Kuwait (5), UAE (3), Oman (1) and multiple countries (2). The most common factors associated with asthma control were: asthma-related education (13 articles), demographics (11articles), comorbidities (11 articles) and environmental exposures (11 articles). Review 2 identified 61 articles reporting ICS/LABA FDC effectiveness studies from countries outside of the GCC. Of these, six RCTs were critically appraised. The adequacy of RCTs in informing clinical practice varied when appraised against previously published criteria.

**Conclusions:**

Asthma-related education was the most recurring factor associated with asthma control in the GCC countries. Moreover, the generalisability of ICS/LABA FDC studies to this region is variable. Hence, asthma patients in the region, particularly those on ICS/LABA FDC, will continue to require physician review and oversight. While our findings provide evidence for local treatment guidelines, further research is required in GCC countries to establish the causal pathways through which asthma-related education influence asthma control for patients on ICS/LABA FDC therapy.

## Background

The global public health burden of asthma is significant, with an estimated global prevalence of 358 million [1]. The Disability Adjusted Life Years (DALYs) attributed to asthma globally across all ages was 23.7 million in 2016 [2]. This chronic condition therefore represents a significant burden to healthcare systems and to individuals living with asthma. While asthma research has received extensive attention worldwide, research on asthma control and the effectiveness of public health interventions is still limited within the Gulf Cooperation Council (GCC) countries (namely Bahrain, Kuwait, Oman, Qatar, Saudi Arabia and the United Arab Emirates [UAE]).

Insight into the prevalence and burden of asthma within the region has been provided by the SNAPSHOT programme [3], a study of large random samples from the general populations of Middle Eastern countries including Kuwait, Saudi Arabia and the UAE (Gulf cluster). The reported prevalence of asthma was 6.4% across all of the assessed Middle Eastern countries and 7.6% within the Gulf cluster [3].

The extent to which the effects of asthma can be seen in individual patients, or reduced by pharmacological or non-pharmacological interventions is known as asthma control [4]. The Epidemiological Study on the Management of Asthma in Asthmatic Middle East Adult Population (ESMAA) re-affirmed the low levels of asthma control in the GCC countries, with the highest levels reported as 41% and 42.6% in Qatar and Kuwait respectively [5].

Numerous risk and prognostic factors have been associated with asthma control [6]. These include genetics, tobacco exposure, occupational exposure, air pollution, respiratory infection, and adherence to treatment [7, 8]. While factors associated with asthma control are generally thought to be similar worldwide, the strength of association may vary both within and, between different populations [9]. Knowledge of local factors associated with asthma control is therefore critical in implementing effective asthma management strategies. Consequently, findings from studies conducted within specific populations of interest, or generalisable to these populations, are vital in informing asthma management. Despite numerous published studies, there is a paucity of evidence synthesis on asthma control factors in the GCC countries. More significantly, evidence relating to the effectiveness of asthma interventions in clinical practice within GCC countries is sparse. Given that the goal of asthma management is to achieve optimal asthma control, it is imperative to provide asthma treatment guideline developers with timely evidence that is relevant to the local population.

Inhaled corticosteroid (ICS) and long-acting beta-agonist (LABA) fixed dose combinations (FDC) are the most reported therapeutic intervention for asthma management in the GCC countries, with a significant proportion of patients in the GCC region being in steps 3 and 4 of the Global Initiative of Asthma (GINA) management guidelines [4, 10]. The place of FDC of ICS and LABA in the management of asthma is also recognized in local treatment guidelines within the GCC, such as the Saudi Initiative for Asthma guidelines [11], the National Asthma Management Guidelines in Oman [12] and the National Clinical Guidelines on the diagnosis and management of asthma in adults by the Qatar Ministry of Public Health [13]. A recent study within the region has shown that patients on ICS/LABA FDCs were more likely to achieve asthma control compared to patients not managed on this therapy [14]. This may provide possible explanation as to why ICS/LABA FDC is the choice of many treating physicians within the region. The use of ICS/LABA in these countries has been reported to range from 73.1% in the UAE to 83.7% in Qatar, with a similar proportion reported in Saudi Arabia (76.3%) and Kuwait (76.8%) [5]. These estimates include instances in which ICS/LABA FDC is either used as the main asthma treatment or in combination with other therapies.

While the efficacy of different ICS/LABA FDCs has been established in traditional randomised controlled trials (RCTs) with strict eligibility criteria, their benefit in actual clinical practice may be reduced by numerous factors associated with asthma control [15]. Many of these factors have been reported in the literature, such as adherence to treatment, comorbidities, polypharmacy and education [16]. The extent to which locally relevant factors are accounted for in effectiveness studies of asthma therapeutic interventions is therefore important when determining the generalisability of evidence from these studies [15]. Moreover, only around 3.3% of patients from actual clinical practice have been found to be eligible for inclusion in traditional RCTs [17].

Studies conducted within the GCC countries are predominantly cross-sectional in nature and have been conducted in different healthcare settings. Most of these studies are descriptive and therefore only estimate the prevalence of asthma and, report factors associated with the disease [10]. While the prevalence of asthma has been informed by the estimated ranges reported in these studies [11], a synthesis of important asthma control factors in these countries will be informative for updates to local asthma management guidelines and practice. Systematic literature reviews (SLRs) are highest in the traditional hierarchy of evidence but few evidences of this type can be found in the region [18–20]. This may be due to several reasons, including the quality and heterogeneity of studies, as well as perhaps the required time commitment of conducting such extensive reviews [21]. Rapid reviews offer a time-sensitive alternative, using abridged SLR methods to generate evidence for healthcare decision makers. These reviews provide evidence summaries that can inform local treatment guidelines and other public health interventions [22, 23]. Moreover, rapid reviews can provide useful evidence, specifically on pharmacological interventions [24]. Such reviews have proven to be useful in certain healthcare settings as they can address gaps in knowledge through provision of reliable, user-friendly and timely evidence [22, 23].

In the present study, a rapid literature review approach was used to address two conceptually sequential objectives: Review 1, to ascertain factors associated with asthma control in the GCC countries (Bahrain, Kuwait, Oman, Qatar, Saudi Arabia and the UAE); and Review 2, to assess the effectiveness of ICS/LABA FDC therapy in asthma control that are generalisable to asthma management in the GCC countries.

## Methods

We conducted two rapid literature reviews in order to provide a time-sensitive summary of factors associated with asthma control in the GCC countries, and ICS/LABA FDC effectiveness studies in asthma to provide evidence for policy and practice of asthma management.

### Search strategy

A facet analysis was conducted on the respective research questions by identifying relevant search terms using the Population, Intervention, Comparator, Outcomes and Study Design (PICOS) framework. Appropriate terminologies were applied in selected bibliographic databases to inform a comprehensive search strategy (Table 1) [25]. The choice of bibliographic databases for this review was informed by the need to ensure the respective literature searches were as extensive as possible thereby reducing the risk of publication bias. PubMed and EMBASE were chosen as they contain indexed abstracts for recent publications [26]. The initial searches were conducted in January 2018 to include publications up to the date of search. As part of an editorial process to bring the literature coverage up to date, updated searches were conducted to include literature up to 4 November 2019. Only search outputs containing abstracts were considered for screening.

**Table 1:** Framework and search strategy for analyses

Review 1			
PICOS	FACET Analysis	Terms (January 2018 Search)	Terms (January 2018 – November 2019 Search)
Population	Patients with asthma	Asthma	Asthma
Intervention	N/A	N/A	N/A
Comparator	N/A	N/A	N/A
Outcome	Factors that are likely to affect asthma control	Adherence, compliance, education, dosing frequency inhaler, age, lifestyle, gender, comorbidities	Adherence, compliance, education, dosing frequency, inhaler, age, lifestyle, gender, comorbidities
Setting	Gulf Cooperation Council countries	Bahrain, Kuwait, Oman, Qatar, Saudi Arabia and United Arab Emirates	Bahrain, Kuwait, Oman, Qatar, Saudi Arabia and United Arab Emirates
LANGUAGE LIMIT	–	EMBASE: English language PubMed: None	EMBASE: English language PubMed: None
TIME LIMIT	–	None	EMBASE: Published between 2018 and 2019 PubMed: Publication between 2018/01/01 and 2019/11/04
Other LIMITS		EMBASE: Studies in humans, publications with abstract. PubMed: None	EMBASE: Studies in humans, publications with abstract PubMed: None
**Review 2**			
Population	Patients with asthma	Asthma	Asthma
Intervention	ICS/LABA	Corticosteroid AND long AND acting AND agonist	
Comparator	N/A		
Outcome	Include all measures of asthma control in the literature	[No term was adopted as it was deemed more appropriate to consider in search screening than to limit by term]	[No term was adopted as it was deemed more appropriate to consider in search screening than to limit by term]
Setting	N/A		
Study design	All study types that may be used in effectiveness research	Effectiveness studies, real world studies, real life, cohort analysis, retrospective studies, database studies	RCTs, Randomised Control Trials, effectiveness studies, real world evidence, real life studies, real world data, real world studies
LANGUAGE LIMIT	English language	Database limit: English language	Database limit: English language
TIME LIMIT	NONE	January 2018	January 2018 – November 2019
Other LIMITS			Human studies: randomised controlled trial, systematic review

*ICS* Inhaled corticosteroid; *ICS/LABA* Inhaled corticosteroid/long-acting beta-agonist; *LABA* Long-acting beta-agonist; *N/A* Not applicable; *PICOS* Population, Intervention, Comparator, Outcomes and Study Design; *RCTs* Randomised controlled trials

### Eligibility of search output

Identified publications were screened for eligibility by title and abstract. Studies were excluded from the search if they were: duplicates, irrelevant to the research question, studying the wrong intervention or outcome, non-empirical studies such as review articles, conference proceedings, guidelines, or conducted in a different base population. Two reviewers conducted the screening, with a third reviewer providing input in instances of discrepancy. Pre-defined information from the full articles of eligible papers was extracted using an extraction grid created by the review team specifically for this purpose.

### Quality assessment

In order to assess the quality of eligible publications, adapted Critical Appraisal Skills Programme (CASP) study checklists were used in the respective reviews [27, 28]. We added questions to the checklist to explore the interrelationships of putative asthma control factors. These questions were: (i) whether all important confounders were identified; (ii) if the method of adjusting for confounding was reported; (iii) if explanatory variables modulating the effect size of asthma therapy interventions were reported; and (iv) if there was a causal web reported or deducible. These questions were added to mobilise additional knowledge about asthma control factors from these studies [29].

The quality assessment of eligible studies in the two reviews comprised of two parts. First was an assessment of the internal validity of individual studies. This included an appraisal of the study objective in relation to the definition of exposure and outcome(s) of interest and statistical analysis conducted, as well as study design and analysis methods employed to reduce biases. The second part was an assessment of generalizability of the study findings; this assessed how findings from each study related to other available evidence, and their implications for asthma management guidelines and practice.

Critical appraisal was not possible in instances where the full article could not be retrieved. Where critical appraisal was conducted, some factors were not included in the cluster analysis when these were considered as proxies of other factors, or mediators of the relationship between other identified factors and asthma control.

### Knowledge-user involvement

Collaboration with a consultant pulmonologist – who is also a co-author of this research and widely published on asthma control in GCC countries – was an integral part of our rapid review process. During the planning and initiation of the rapid review, his expert contributions included: insights on putative asthma control factors of clinical significance in the GCC countries; expert opinion on reported factors in the context of local asthma management practices; eliciting the broader opinions of experts during the presentation of preliminary results at a scientific congress; and the translation of wider knowledge to the findings, such as the review of recent updates to treatment guidelines. Data analysis was informed by the Cochrane Consumers and Communication Review Group guide on data synthesis and analysis [26]. The study results were reported using the Preferred Reporting Items for Systematic Reviews and Meta-Analysis (PRISMA) guidance [30].

### Asthma control factors in the GCC countries – review 1

**Fig. 1:**
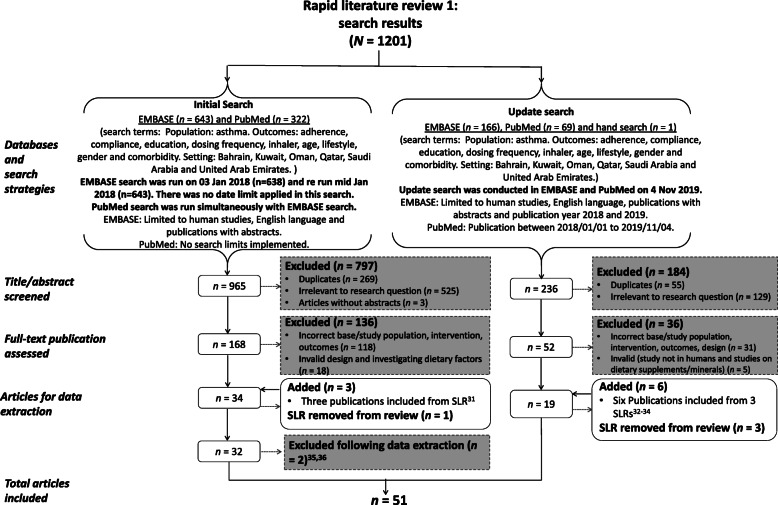
Asthma control factors in the GCC countries (Review 1): Flow diagram of search strategy/approach. SLR: Systematic Literature Review; GCC: Gulf Cooperation Council [31–36]

The search strategy and article eligibility considerations were guided by the PICOS framework (Table 1) - presented as a flow diagram in Fig. 1. Articles were included if they reported univariate or multivariate analysis of asthma control factors in Bahrain, Kuwait, Oman, Qatar, Saudi Arabia or UAE. The CASP Cohort Study checklist was utilised for the asthma control factors literature critical appraisal [27].

#### Cluster analysis in review 1: asthma control factors

Clinically significant asthma control factors were identified in eligible studies if they were statistically significant with reported precision (i.e. confidence interval) in multivariate analysis, or statistically significant with precision in univariate analysis where multivariate analysis was not reported. Using a similar approach to a previously reported study, a clustering method was used to summarise the different factors reported in the literature [37]. As asthma control factors reported in individual publications could be multiple and therefore eligible to be summarized in multiple clusters, these clusters are independent of the number of studies reviewed. Principal summary measures were counts of the number of each cluster reported in the literature. The resulting clustered factors were presented using bar graphs to illustrate the frequency of asthma control factors in the different settings in which they were studied.

### Effectiveness studies (RCTs) of ICS LABA – review 2

Our first step after conducting the literature search was to identify analytical studies that empirically sought to demonstrate causal relations between ICS/LABA FDCs and asthma control. This was achieved by extracting information available on the study design of individual studies and included information on the intervention and comparator (where applicable), effect measure, sample size and analysis of confounding. Published opinions and other materials that were not empirical studies or not relevant to the review question were excluded. Retained publications were predominantly observational studies. Due to the wide spectrum of retrieved observational studies, and the widely accepted view on the robustness of RCTs over observational studies in analytical research, we limited our analysis to RCTs [38]. However, given the review question, there was the need to distinguish between conventional efficacy RCTs and effectiveness RCTs by adopting previously published criteria [39]. The adapted CASP Randomised Control Trials checklist was used to appraise the quality of the eligible publications [28]. Data were summarised descriptively.

## Results

### Asthma control factors in the GCC countries (review 1)

In total, our targeted literature search returned 1201 articles. Of these, 51 articles met the pre-defined eligibility criteria and were retained for critical appraisal and analysis (Fig. 1). Of the eligible literature, the earliest identified publication on asthma control factors in the GCC countries was published in 1990. Over 74% (38 articles) were published between 2010 to date of this review. Thirty-five articles reported studies conducted within Saudi Arabia, with three articles from the UAE, five each from Qatar and Kuwait and one from Oman; two articles reported a study conducted across multiple countries. No articles reporting studies conducted in Bahrain were identified.

#### Quality appraisal

The majority (*n* = 44) of eligible study publications addressed a clearly focused research question. Most of the studies across the GCC countries were conducted in convenient samples of patients with asthma (i.e. using a non-probability sampling method where the study sample was derived from easy-to-reach patients). Only eight of the studies included all patients within the sample frame or utilised probability sampling methods. Exposure to factors of asthma control was accurately measured in 42 of the identified articles, while asthma control was measured using a validated tool in 27 studies. Based on the study reports, only 22 studies identified all important confounders and 18 adjusted for confounders in their study design and/or analysis. Based on the reported information about variables collected from study participants at different time points, the follow-up of study participants was assessed as complete in 30 studies and of long duration in 10 studies (i.e. studied over a minimum of 1 year to account for variations in temporal factors such as seasonality). A total of 36 studies reported some measure of result precision (mostly confidence intervals). Most studies (*n* = 41) did not report any form of effect modification and almost the same number of studies (*n* = 40) did not report any mediator variable.

#### Cluster analysis

**Fig. 2:**
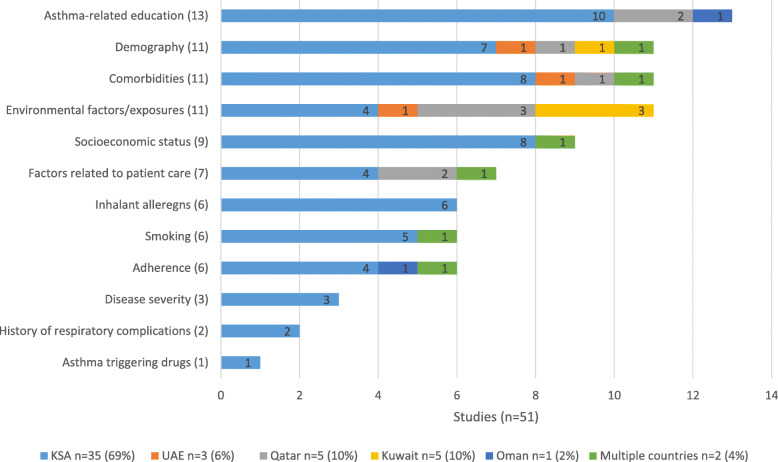
Asthma control factors in the GCC countries (Review 1): Total number of articles by country. GCC: Gulf Cooperation Council; UAE: United Arab Emirates reported; *n* = total number of articles representing results from country; bar counts number of reports from each country, some articles reported multiple asthma control factors; Bahrain was assessed per method but provided no reports. One article from Kuwait did not report any factor cluster

**Fig. 3:**
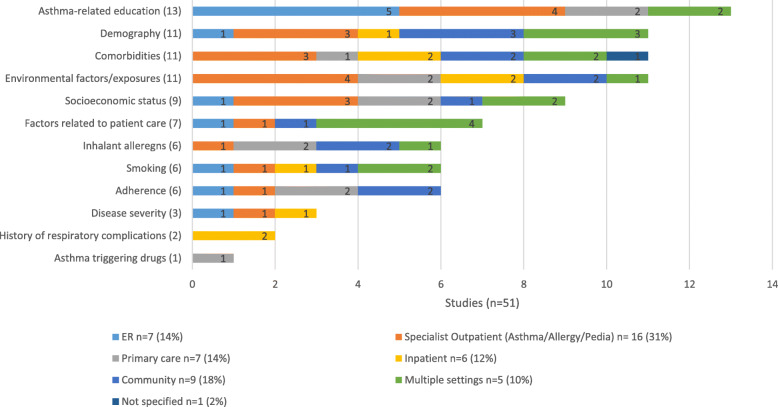
Asthma control factors in the GCC countries (Review 1): Total number of articles by setting. ER: Emergency room; GCC: Gulf Cooperation Council; *n* = total number of articles representing results from each setting; bar counts number of reports from each setting. Some articles reported multiple asthma control factors

**Fig. 4:**
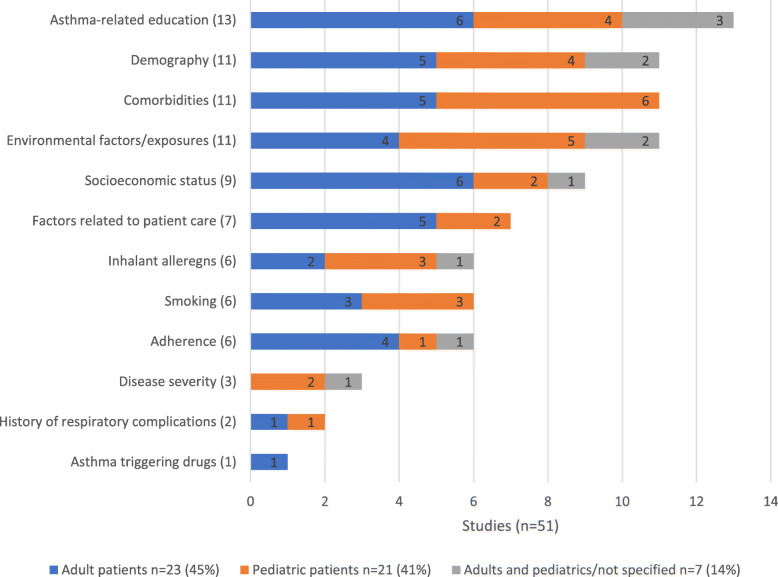
Asthma control factors in the GCC countries (Review 1): Total number of articles by age classification (adult and paediatric). *n* = total number of articles results from each setting; bar counts number of reports for each age group

Factors identified in the literature of asthma control in GCC countries were varied (Table 2). Following critical appraisal of eligible publications, the most recurring asthma control factor cluster was asthma-related education (*n* = 13). This was followed by demography, comorbidities and environmental factors clusters (*n* = 11 respectively). Other asthma control factors clusters were socioeconomic status (*n* = 9) and factors related to patient care (*n* = 7). Inhalant allergens, smoking and adherence to treatment were equally recurrent (*n* = 6 respectively) (Fig. 2). The least recurring asthma control factors were disease severity (*n* = 3), history of respiratory complications (*n* = 2) and asthma-triggering drugs (*n* = 1). The most represented country in the analysis was Saudi Arabia (35 publications). Other countries on which published literature on asthma control factors were identified were Qatar (5 publications), Kuwait (5 publications) and Oman (1 publications) (Fig. 2). The recurrence of asthma-related education factors associated with asthma control varied between Saudi Arabia (*n* = 10), Qatar (*n* = 2) and Oman (n = 1). Demography, comorbidities and environmental exposures were associated with asthma control in the four GCC countries in which eligible published studies were identified (Kuwait, Qatar, Saudi Arabia and UAE). The remaining factor clusters that were significantly associated with asthma control were exclusively identified from studies conducted in Saudi Arabia, namely: asthma triggering drugs, history of respiratory complications, disease severity, and inhalant allergens. Socioeconomic status was mostly reported in Saudi Arabia as a cluster of asthma control factor (Fig. 2). The most common setting in which studies on asthma control were conducted were specialist outpatient clinics (*n* = 16), followed by community settings (e.g. schools) (*n* = 9) and primary care (*n* = 7) (Fig.3). Of the 16 articles reporting results from specialist outpatient clinics, the most commonly reported clusters associated with asthma control were asthma-related education factors (*n* = 4) and environmental factors/exposure (*n* = 4). When assessed against healthcare setting, asthma-related education cluster was the most recurrent asthma control factor in the Emergency Room setting (ER) (*n* = 5). In the specialist outpatient setting, asthma-related education (*n* = 4) and environmental exposures (n = 4) were most recurrent asthma control factors clusters. Demography (*n* = 3), comorbidities (n = 3) and socioeconomic status (*n* = 3) were also often recurring factors associated with asthma control in the specialist outpatient setting. In the primary and community care settings, environmental factors (n = 4), adherence (n = 4) and inhalant allergens (n = 4) were the most recurrent asthma control factors. Other factors associated with asthma control in the primary and community care settings are demography (n = 3), comorbidity (n = 3) and socioeconomic status (n = 3). The most recurring factors associated with asthma control in inpatient care included comorbidities (*n* = 2), environmental exposure (n = 2) and history of respiratory complications (n = 2). Of the eligible publications on asthma control factors in the GCC countries, 23 studies (45%) were conducted in adult patient populations only, while 21 studies (41%) were conducted in paediatric patient populations only (Fig. 4). Difference was seen in the recurrence of socioeconomic status cluster as being associated with asthma control in adults [6] compared to paediatric population [2]. Similarly, factors cluster of patient care was more recurrent in studies conducted in the adult patient population [5] compared to the paediatric asthma population [2]. Furthermore, adherence to asthma treatment was more often associated with asthma control in adult patients [4] than in paediatric patients [1]. Comorbidities, including disability, in the adult population studies (*n* = 5) recurred less to the paediatric population studies (*n* = 6). (Fig. 4).

**Table 2:** Asthma control factors in the GCC countries (Review 1): Factor clusters identified during literature analysis

Factor Clusters	Constituent Factors Associated with Asthma Control
Asthma-related education	Education about: asthma, asthma medicines, correct use of inhaler device, how to prevent and treat symptoms, perception on the role of ICS, perception on using ER for asthma care
Environmental factors/exposures	Altitude, dust, air pollution, seasonal variations, sandstorms, workplace triggers, thunderstorms, broken mountains, temperature, atmospheric pressure, incense, wood smoke, household chemicals, soft drinks consumption
Comorbidities	Psychiatric illness (anxiety/depression/stress), allergies (rhinitis, sinusitis, skin allergy), family history of allergy, GERD, obesity, disability (including work-related disability), respiratory pathogens
Disease severity	Actual/perceived disease severity, multiple ER visits/hospital admissions, previous requirement of systemic steroids
Demographic factors	Age, gender, geographical distribution, number of siblings, number of pregnancies, marital status, nationality, residence (urban/rural)
Smoking	Smoking (active and passive)
Asthma triggering drugs	ACE inhibitors, β-blockers, aspirin, and NSAIDs
Adherence	Adherence/regular ICS use, concomitant use of prophylactic medicines
Socioeconomic status	Level of Education, Household income, occupation, employment status, socioeconomic class, bedroom sharing, daily stress
Factors related to patient care	Presence of asthma management protocol, level of physician care, regular follow-ups, medical insurance, time from diagnosis, length of post-delivery hospital stay, use of herbal medicine
Inhalant allergens	House dust mite, cat epithelia, cockroaches, moulds, unsealed mattresses, bedroom carpets, pets, rye wheat, pollens
History of respiratory complications	Previous ICU/Neonatal ICU admission, bronchopulmonary dysplasia, and history of previous asthma-related hospital admissions, tracheoesophageal fistulae, recurrent aspirations, intubation, intravenous steroids

*ACE* Angiotensin-converting enzyme; *ER* Emergency room; *GCC* Gulf Cooperation Council; *GERD* Gastroesophageal reflux disease; *ICS* Inhaled corticosteroid; *ICU* Intensive care unit; *NSAID* Nonsteroidal anti-inflammatory drug

Similar factors were grouped into the above clusters

### Effectiveness studies (RCTs) of ICS/LABA FDC (review 2)

**Fig. 5:**
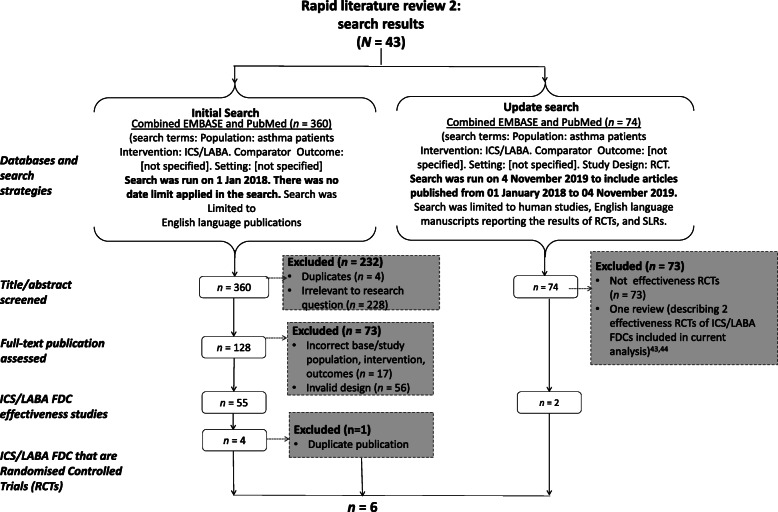
Effectiveness studies of ICS LABA FDC (Review 2): Flow diagram of search strategy/approach. FDC: Fixed-dose combination; ICS: Inhaled corticosteroid; LABA: Long-acting beta-agonist; RCT: Randomised controlled trial. [43, 44]

In total, our targeted literature search identified 435 articles in the EMBASE and PubMed databases. Of these, 61 articles were identified as publications of effectiveness studies of ICS/LABA FDC, i.e. conducted in broad patient populations with conditions close to actual clinical practice (Fig. 5). The earliest year of publication of these studies was in 2004, with the latest publication in 2017. The designs of these studies were variable and included historical cohort studies (*n* = 39), prospective cohort studies (*n* = 8), RCTs (*n* = 6) and cross-sectional studies (*n* = 3). Only the six effectiveness RCTs were retained for quality appraisal and the extent to which these studies were distinguished as effectiveness RCTs was described (Table 3). The earliest date of publication among the six effectiveness RCTs was in 2010 and the latest publications were in 2019.

**Table 3:** Effectiveness studies (RCTs) of ICS/LABA FDC (Review 2): Assessment of identified effectiveness RCT studies

Effectiveness RCTs	ICS/LABA FDC interventions	Primary care setting?	Broadness of eligibility criteria	Duration	Sample size calculation [sample size, n]	Asthma control?	Safety measure?	ITT population?
Usmani et al. 2017 [40] [NCT02388373]	**Fluticasone propionate /formoterol vs fluticasone propionate /salmeterol**	YES [general practice]	Control or partially controlled with prescription for at least 6 months with no exacerbation in the 3 months prior to enrolment	24 weeks	YES [225]	YES [ACQ7]	YES [Adverse events]	YES [Not as initially planned]
Woodcock et al. 2017 [41] [NCT01706198]	**Fluticasone furoate/vilanterol vs usual care including ICS/LABA FDC**	YES [general practice]	GPs’ diagnosis of symptomatic asthma and on maintenance inhaler therapy	52 weeks	YES [4233]	YES [ACT]	YES [Adverse events]	YES
Aubier et al. 2010 [42] [NCT00463866]	**Two budesonide/formoterol maintenance doses**	Not indicated	Moderate-to-severe asthma who were symptomatic despite daily use of an ICS with or without LABA	6 months	YES [8424]	NO [Time to severe exacerbation]	YES [Adverse events]	Not Indicated
Beasley et al. 2019 [43] [ACTRN12615000999538]	**Budesonide/formoterol 200 μg/6 μg as needed compared to budesonide (200 μg, one inhalation twice daily) plus as needed albuterol or as needed albuterol**	No [primary and secondary care]	Self-reported doctor diagnosis of asthma and use of a SABA as the sole asthma therapy in the previous 3 months on ≥2 occasions, but on an average of ≤2 occasions per day, in the previous 4 weeks	52 weeks	YES [675]	YES [ACQ5]	YES [Adverse events]	YES
Hardy et al. 2019 [44] [ACTRN12616000377437]	**Budesonide/formoterol 200 μg/6 μg reliever therapy compared to maintenance budesonide plus terbutaline**	No [primary care or hospital-based clinical trials units and primary care practices]	Self-reported doctor diagnosis of asthma and taking either SABA reliever therapy alone or SABA plus low to moderate dose ICS in the 12 weeks before randomisation	52 weeks	YES [890]	YES [ACQ5]	YES [Adverse events]	YES
Hozawa et al. 2014 [45]	**Budesonide/formoterol for maintenance and reliever therapy and fluticasone propionate/salmeterol**	No [outpatient]	Inadequately controlled asthma patients treated with a medium dose of ICS alone and using a SABA 2–6 occasions/ week	8 weeks	Not Indicated [30]	NO [change in FeNO]	YES [Adverse events]	NO

*ACQ* Asthma Control Questionnaire; *ACT* Asthma Control Test; *AHRQ* Agency for Healthcare Research and Quality; *FDC* Fixed-dose combination; *FeNO* Fractional exhaled nitric oxide; *GP* General practitioner; *ICS* Inhaled corticosteroid; *ITT* Intention-to-treat; *LABA* Long-acting beta-agonist; *RCT* Randomised controlled trial; *SABA* Short-acting beta-agonist

#### Quality appraisal

All RCT studies appraised in this analysis addressed clearly defined questions and assigned patients to treatment groups by randomisation. Based on published information, five of the six studies had treatment groups that were similar to each other at the start of each trial. In one study, the authors reported that patients randomised to experimental ICS/LABA FDC were more likely to revert to their original treatment. Unlike traditional RCTs, study investigators were not blinded in any of the appraised studies – this was expected due to the studies being conducted in conditions similar to actual clinical practice.

The treatment effect of ICS/LABA FDC reported in the appraised studies included : difference in means in Asthma Control Questionnaire 7 (ACQ7) [40] odds ratios, where changes in Asthma Control Test (ACT) scores were assessed [41]; hazard ratios where time to asthma exacerbation was assessed [42]; and relative rates, where the number of exacerbations per patient per year was reported [43, 44]. One study measured changes in fractional exhaled nitric oxide (FeNO) values from baseline [45]. All but one of the studies [45] reported 95% confidence intervals to indicate the precision of the treatment effect. In one of these studies, the 95% confidence interval for the primary outcome measure included the reference value of 1 [44].

There were variable limitations in the generalisability of the identified ICS/LABA FDC effectiveness RCTs to the local population in GCC countries. Reasons ranged from limitations in study design to inferences from studies on clinical practice. Hozawa et al. [45] had a limited study duration (8 weeks) and excluded patients with a history of smoking, thus limiting the generalisability of the study outcomes. Usmani et al. [40] acknowledge that the study design might have contributed to the differential rate of non-completion and missing outcomes. Lack of clarity on identifying patients suitable for step-down therapy in routine daily practice [43], and the implication of potential sub-optimal control in patients who may request as-needed therapy [44], were some other factors that limited the application of other study findings to clinical practice.

Two key trends emerged concerning the outcome measure of the different ICS/LABA FDC studies. The first was the benefit observed in improving asthma control factors, which is the main goal of asthma management. The second was the benefit reported in reducing the number of asthma exacerbations.

#### Assessment of identified effectiveness RCT studies against effectiveness studies criteria

Four unique RCTs were identified during the initial literature search and two additional eligible studies were identified during the updated search, taking the total number of identified publications reporting the results of effectiveness RCTs to six. The six ICS/LABA FDC effectiveness RCTs had different comparator groups, such as different doses of the same ICS/LABA FDC, alternative ICS/LABA FDC, and usual care (including other therapeutic options).

Most studies recruited patients from primary care settings which suggests that the broadest spectrum of asthma patients, within the healthcare systems in which the studies were conducted, are present in this setting. Study setting was not reported in one study [42]. Patient eligibility criteria varied across all the studies. The broadest eligibility criteria was in Woodcock et al. [41], in which patients required a general practitioner diagnosis of symptomatic asthma and use of maintenance inhaler therapy. Stricter eligibility criteria across studies included stipulation of a 6-month exacerbation-free period [40] and moderate-to-severe asthma [42]. Two studies adopted eligibility criteria which required patients to have a self-reported doctor diagnosis of asthma and use of a short-acting beta-agonist (SABA) alone [43, 44] or in combination with low-to-moderate dose ICS [44]. The duration of the patient observation period also varied amongst the studies. The shortest study duration was 8 weeks [43] and the longest was 52 weeks [41, 43]. Usmani et al. [40] had a study observation period of 24 weeks, while Aubier et al. [42] had an observation period of 6 months. Sample size calculations and sample sizes were reported in all but one study [43]. Asthma control was measured by either ACT or ACQ7 in four out of six studies. The two studies that did not assess asthma control measured rate of, and time to severe exacerbation [42] and, change in FeNO respectively [45]. The intent-to-treat analysis was reported in four studies [40, 41, 43, 44] while safety was assessed in all of the studies.

## Discussion

We conducted this dual rapid review to provide timely evidence to inform asthma management guidelines and practice within the GCC countries. The local populations in the GCC countries share a common geographic area known as the Arab peninsular. While anthropology studies have suggested some genetic heterogeneity, there are common features among local populations within the GCC countries [46]. An assessment of local asthma control factors was conducted and, how generalisable the evidence on ICS/LABA FDC effectiveness is, given that this therapeutic approach remains the most prevalent in the GCC countries. Our search identified studies from the GCC countries that reported association between asthma control and the factors contributing to it. These studies varied in their design; the majority of which were cross-sectional. Most studies identified were descriptive and were conducted in Saudi Arabia. This was anticipated given that Saudi Arabia has the largest landmass of the GCC countries [47]. The disproportionate number of eligible publications on asthma control factors in the GCC countries up until 2018 (*n* = 32) and after 2018 (*n* = 19) may be due to several reasons. Notable among the reasons is the significant increase in medical institutions [48] and academic research [49] in the GCC countries within a relatively short period of time.

Our search strategy for the review on asthma control factors was developed to be as broad as possible. Our preliminary scoping search had revealed that many asthma related studies published in the region, particularly those reporting on prevalence, also reported on asthma control factors. Identified studies were generally lacking analytical framework to inform on the actual role of factors beyond association. For example, a number of factors reported in the literature may have been mediators or proxies of other putative factors not directly measured [50]. This limited our ability to ascertain temporal components of asthma control and also, therefore, to sometimes distinguish between risk and prognostic factors [51].

Of all the factors reported to be associated with asthma control within the GCC countries, asthma-related education recurred most often. These encompassed: education about asthma, asthma medicines and correct use of inhaler device; how to prevent and treat symptoms; and perceptions on the role of ICS and emergency room use for asthma care. While studies within GCC countries did not delineate between formal and informal approaches to asthma-related education, a systematic review of studies of educational interventions in asthma management found that delivery methods used in asthma education interventions were mostly informal [52]. The review also reported that few of these asthma-related education interventions were home or community based. Education factors relating to patient knowledge about their symptoms, perceptions about their medications, and correct use of their device are all areas within the scope of the healthcare professional-patient relationship [53]. Consequently, the lack of review and physician oversight is one of the contributors to poor asthma control and may lead to over-reliance on rescue medication. Studies from other parts of the world have shown that only around 50% of patients claim to be able to manage their asthma or have good knowledge about asthma treatment and adherence to controller medication [54–56]. Hence, it may not be appropriate for patients to judge their own symptoms and take decisions on their therapies without guidance from an appropriately trained healthcare professional [55].

For non-pharmacological interventions, our findings suggest that significant improvements to asthma management may help to better address asthma-related factors and therefore achieve optimal asthma control in the region. One such asthma management practice is the provision of asthma educators in all healthcare settings thereby filling the void due to time constraints of asthma treating physicians [57].

Other factors associated with asthma control included demography, comorbidities, environmental factors, factors related to patient care, smoking, adherence, inhalant allergens and disease severity. As these clusters are likely to be interrelated, modifying the most distal in the interrelationships of these factors may alleviate asthma control due to their influence on multiple and more directly associated factors. Examples of distal factors in chronic diseases include education and socioeconomic factors [58]. Further research is required to establish the interrelationships of these factors as this is important when developing public health interventions through multi-disciplinary approaches to asthma management.

Our review of ICS/LABA FDC effectiveness studies was insightful. Categorising ICS/LABA FDC studies as either efficacy or effectiveness studies is best considered as a spectrum. We explored this spectrum by using published criteria to provide an objective assessment of how well each of the studies could inform local clinical practice.

In the earlier published studies of ICS/LABA FDCs, lung function, symptoms, use of rescue medication and adverse events were most frequently reported. However, there were differences in the individual trials which meant that few comparisons could be made [59]. This necessitated comparative studies in broad asthma patient populations within clinical practice. The first prospective, Phase III, pragmatic randomised controlled trial (RCT) conducted in conditions close to clinical practice, was reported in 2017 [60]. Healthcare professionals and payers continue to be in need of such studies for treatments that have newly gained Marketing Authorisation (MA) [61]. Given the few ICS/LABA FDC effectiveness RCTs identified, it was not surprising that none were from GCC countries. It was surprising, however, that our initial search output – which mostly comprised of traditional efficacy RCTs – included very few studies conducted in the GCC countries. The possible reasons for this are beyond the scope of our review. Nevertheless, this finding indicates that healthcare professionals and treatment guideline developers in the region will continue to rely on evidence presented in studies conducted elsewhere in order to make local evidence-based decisions on asthma management. As such, it is important to assess the generalisability of such evidence to local clinical practice and the appropriateness of its influence on local asthma management guidelines and practices of treating physicians within the GCC countries.

Despite identifying few (six) ICS/LABA FDC effectiveness studies, there was heterogeneity in how these studies met the applied criteria [40–45]. A key consideration in effectiveness studies is ensuring that the study population is broad enough to be representative of the target patient population. Patient eligibility criteria in the studies ranged from controlled to partially controlled patients, as in the case of Usmani et al. [40], to moderate-to-severe asthma in the case of Aubier et al. [42]. The eligibility criteria adopted in Usmani et al. [40] was therefore broader than that applied in Aubier et al. [42]. Other studies defined patients simply by diagnosis of asthma. Woodcock et al. [41] defined eligible patients as those with a general practitioner diagnosis, which was based on medical records. This was likely to be more reliable than the self-reported doctor diagnosis adopted by Beasley et al. [43] and Hardy et al. [44], and may explain some of the criticisms of the latter two studies [62]. In the study of Beasley et al. [43], as-needed budesonide-formoterol reduced the number of exacerbations compared to SABA alone, but the study was conducted in a population in which SABA alone was not indicated (GINA step II) [4]. In the case of Hardy et al. [44], their study of budesonide-formoterol reliever therapy compared to maintenance budesonide plus terbutaline may not have been conducted in patients with mild asthma only, as it was apparent that some moderate asthma patients were included based on the treatments taken.

With respect to study duration, Usmani et al. [40] acknowledged that the 12-week follow-up period in each phase of their study of fluticasone propionate/formoterol vs fluticasone propionate/salmeterol was likely to have been insufficient to capture seasonal variations in asthma control and exacerbations. This is particularly important in the GCC countries given the effect of seasonality on asthma control. In contrast, Woodcock et al. [41] showed that fluticasone furoate/vilanterol significantly improved asthma control compared with usual care, including ICS/LABA combinations, over a 52-week study. Aubier et al. [42] (6 months observation period) and Hozawa et al. [45] (8 weeks observation period) are therefore likely to have had insufficient patient observation period – though neither study discussed this.

There were significant differences in the sample sizes of the effectiveness RCTs of ICS/LABA combinations identified in the review. Usmani et al. [40] acknowledged that one of the limitations of their study, in which they assessed change to and step-down from fluticasone propionate/formoterol, was the small sample of patients (*n* = 225) studied. This meant study power was limited for detecting the predictors of response to step-down therapy. With a total of 8424 study patients, Aubier et al. [42] calculated the sample size required for each group to detect a reduction in the proportion of patients experiencing a severe asthma exacerbation. The study by Woodcock et al. [41], with 4233 patients, was reported to be the largest randomised, comparative effectiveness study conducted in a population intended to represent that seen in everyday clinical practice. This study was powered to detect relative improvements in asthma control. Both Beasley et al. [43] and Hardy et al. [44] conducted sample size calculations and studied 675 and 890 patients respectively.

Measurement of asthma control, which is the main goal of asthma management, also varied in the studies. Different tools were employed to assess asthma control. Usmani et al. [40] suggested that inappropriate assessment of asthma control might contribute to overtreatment. As measured by the ACQ7, asthma control was similar between the fluticasone propionate/formoterol (1000/40 μg) and fluticasone propionate/salmeterol (1000/100 μg) groups; however, a significantly higher percentage of patients were controlled on fluticasone propionate/formoterol (1000/40 μg) when control was defined according to GINA [42]. However, the view of the authors was that no standardised questionnaire exists for assessment of asthma control according to GINA. Aubier et al. [42] reported no significant effect of the maintenance dose of budesonide/formoterol on the risk of exacerbation for patients with a post-bronchodilator peak expiratory flow value ≥80% predicted normal who comprised two-thirds of the population studied. The authors acknowledged that it was unexpected that a low peak expiratory flow value was the only variable predicting response to the higher maintenance dose of budesonide/formoterol. This could suggest that asthma outcomes other than time-to-first exacerbation are more relevant when assessing asthma patients in actual clinical practice. In Hozawa et al. [45], the authors were of the view that FeNO represents airway inflammation. However, this outcome measure may be of very limited utility in the GCC countries.

Our dual rapid reviews had some limitations. We were unable to ascertain how comprehensive our two independent review searches were given the abridged SLR method utilised. Given our search strategy, it is probable that most of the published literature on asthma control factors in the GCC countries was identified. While our search on ICS/LABA FDC effectiveness was also comprehensive, it is possible that our initial assessment in determining effectiveness studies from the search output may have excluded certain publications of RCTs that their authors would consider to be closer to an effectiveness than efficacy study.

In our review approach we relied mostly on published and readily available information – we did not consult authors to enquire about any unpublished analysis addendum to their papers. Hence, any established relationship of the associated factors of asthma control may not have been considered in our review. The quality of some of the evidence considered, particularly for the review on asthma control factors in the GCC countries, varied considerably. The overall utility of studies on asthma control factors in our analysis was evaluated in view of the overall implication for practice and was also informed by the insights of our expert knowledge user.

Rapid reviews can provide data needed to address issues such as health system policies and effectiveness. The World Health Organisation and Cochrane have acknowledged the role of rapid reviews in building evidence-based policies and practices [23]. A major strength of our review, which conforms to the World Health Organisation recommendations on rapid literature reviews, is that our research team included the end-user (a Consultant Pulmonologist representing those who would ultimately use the resulting evidence in clinical practice). Our clinical expert was consulted at all stages of the review process, providing input on review planning and initiation, conduct during the review, and assessment of results. This provides an important insight into the utility of our review. Moreover, the interdisciplinary team involved (including health outcomes scientist, epidemiologist, and pharmacist) enriched the review and its output.

## Conclusion

Within the GCC countries, it is important that the effectiveness of ICS/LABA FDC is established in the context of local asthma control factors. This means that for evidence to be generalisable to the region, the studies from which evidence is generated must account for the asthma control factors pertinent in the region. This is achieved when studies are designed to minimise bias and adjust for putative factors associated with asthma control. Asthma-related education was the most recurrent cluster associated with asthma control in the GCC countries. Education factors that relate to patient knowledge about their symptoms, perceptions about their medications, and correct use of their device require physician oversight to ensure patients’ asthma control and, reduce over-reliance on rescue medication. These findings limit the local relevance of approaches that leave most asthma patients to judge their symptoms for taking medication. This finding is particularly important in the GCC countries where most patients studied have been reported to be on ICS/LABA FDCs.

Other factors identified as associated with asthma control in our study included demography, comorbidities, environmental factors, factors related to patient care, smoking, adherence, inhalant allergens and disease severity. These factors re-iterate the need for a multi-disciplinary approach to asthma management in the GCC countries.

Generalisability of evidence on the most administered therapeutic intervention in asthma management in the GCC countries requires that the studies are conducted in broad patient populations and in conditions close to actual clinical practice – as assessed by the effectiveness criteria utilised in our review. As our review of ICS/LABA FDC effectiveness studies highlights, the extent to which they can be considered generalisable to the GCC countries to influence treatment guidelines and clinical practice is variable.

## Data Availability

The datasets used and/or analysed during the current study are available from the corresponding author on reasonable request.

## Abbreviations

ACQ7: Asthma Control Questionnaire 7
ACT: Asthma Control Test
CASP: Critical Appraisal Skills Programme
FDC: Fixed dose combinations
FeNO: Fractional exhaled nitric oxide
GCC: Gulf Cooperation Council
GINA: Global Initiative for Asthma
ICS: Inhaled corticosteroid
LABA: Long-acting beta-agonist
PICOS: Population, Intervention, Comparator, Outcomes and Study Design
RCT: Randomised controlled trials
SABA: Short-acting beta-agonist
SLR: Systematic Literature Reviews
UAE: United Arab Emirates
